# The Genetic Architecture of Parallel Armor Plate Reduction in Threespine Sticklebacks

**DOI:** 10.1371/journal.pbio.0020109

**Published:** 2004-03-30

**Authors:** Pamela F Colosimo, Catherine L Peichel, Kirsten Nereng, Benjamin K Blackman, Michael D Shapiro, Dolph Schluter, David M Kingsley

**Affiliations:** **1**Department of Developmental Biology and Howard Hughes Medical Institute, Stanford University School of MedicineStanford, CaliforniaUnited States of America; **2**Fred Hutchinson Cancer Research Center, SeattleWashingtonUnited States of America; **3**University of British Columbia, VancouverBritish ColumbiaCanada

## Abstract

How many genetic changes control the evolution of new traits in natural populations? Are the same genetic changes seen in cases of parallel evolution? Despite long-standing interest in these questions, they have been difficult to address, particularly in vertebrates. We have analyzed the genetic basis of natural variation in three different aspects of the skeletal armor of threespine sticklebacks *(Gasterosteus aculeatus):* the pattern, number, and size of the bony lateral plates. A few chromosomal regions can account for variation in all three aspects of the lateral plates, with one major locus contributing to most of the variation in lateral plate pattern and number. Genetic mapping and allelic complementation experiments show that the same major locus is responsible for the parallel evolution of armor plate reduction in two widely separated populations. These results suggest that a small number of genetic changes can produce major skeletal alterations in natural populations and that the same major locus is used repeatedly when similar traits evolve in different locations.

## Abstract 2

In stickleback populations separated over 10,000 years ago, dramatic morphological evolution appears to result from a relatively small number of genetic changes

## Introduction

The number and type of genetic changes that control morphological and physiological changes during vertebrate evolution are not yet known. The evolutionary history of threespine sticklebacks *(Gasterosteus aculeatus)* provides an unusual opportunity to directly study the genetic architecture of adaptive divergence in natural populations. At the end of the last ice age, marine sticklebacks colonized newly formed freshwater environments throughout the Northern Hemisphere. Over the last 10,000 to 15,000 years, these fish have adapted to a wide range of new ecological conditions, giving rise to diverse populations with striking differences in morphology, physiology, and behavior (Bell and Foster 1994). Major changes in the bony armor have evolved repeatedly in different locations, and several hypotheses have been proposed to explain this morphological transformation, including response to changes in calcium availability (Giles 1983), stream gradients (Baumgartner and Bell 1984), or temperature, salinity, or other factors that may vary in parallel with climate (Heuts 1947; Hagen and Moodie 1982); or exposure to different types of predators (Hagen and Gilbertson 1973a; Moodie et al. 1973; Reimchen 1992; Reimchen 1995).

**Figure 1:**
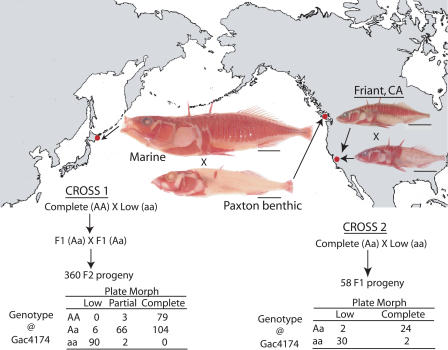
Mapping the Genetic Basis of Lateral Plate Reduction in Different Natural Populations of Threespine Sticklebacks Crossing a completely plated Japanese marine fish with a low-plated fish from Paxton Lake, British Columbia, produced a mixture of complete, partial, and low morph phenotypes in F2 progeny animals (Cross 1). In contrast, crossing a completely plated fish and a low-plated fish from an inland freshwater stream in Friant, California, produced only complete and low-plated progeny (Cross 2). Red dots show the geographic origins of the populations studied. Scale bars equal 1 cm. *AA*, *Aa*, and *aa* refer to genotypes at *Gac4174* (a microsatellite marker) near the major plate locus on LG 4. The genotype at *Gac4174* is missing in ten of the 360 F2s in Cross 1. All fish were stained with alizarin red to reveal bony structures.

Three distinctive patterns of body armor, now known as the “lateral plate morphs,” have been recognized as one of the most distinguishing characteristics in sticklebacks since at least the early 1800s (Cuvier and Valenciennes 1829). Most marine sticklebacks have a continuous row of bony plates that covers the lateral side of the body from head to tail (the “complete morph”; see marine fish in Figure 1). In contrast, many freshwater sticklebacks show substantial reductions in total plate number, developing either as “partial morphs,” which lose plates in the middle of the row (not shown), or as “low morphs,” which retain only a few plates at the anterior end (see Paxton benthic and Friant California [lower animal] fish in Figure 1). The anterior plates present in low morphs are the first to form during larval development. In contrast, the middle plates absent in partial morphs are the last to form during normal development (Igarashi 1964; Igarashi 1970; Bell 1981). Thus, the adult plate patterns of low and partial morphs resemble early developmental stages of plate patterns in complete morphs, and paedomorphosis has been proposed as a possible explanation for the repeated evolution of low and partial morphs from completely plated ancestors (Bell 1981).

This dramatic variation in lateral plate patterning has led to repeated efforts to determine the genetic basis of the major plate morphs. Previous studies have shown that plate morphs are reproducibly inherited in the laboratory and that crosses between different morphs generate relatively simple ratios of the three major phenotypes among the progeny. Based on these qualitative results, at least six different genetic models have been proposed for lateral plate patterns in sticklebacks. The simplest models proposed a single major locus with alternative alleles *(A* and *a)* (Munzing 1959; Avise 1976). The *A* allele was first proposed to be incompletely dominant to the *a* allele, generating either complete *(AA),* partial *(Aa),* or low-plated *(aa)* fish (Munzing 1959). In other populations, the *A* allele may be completely dominant to the *a* allele, producing either complete *(AA, Aa)* or low-plated *(aa)* fish, but no partials (Avise 1976). More complicated models have proposed two major loci controlling plate inheritance (with alternative alleles *A, a* and *B, b*). In one of these models, both major loci contribute equally to plate phenotype, and the total number of *A* and *B* alleles determines whether fish develop as complete (three or more *A* or *B* alleles), partial (two *A* or *B* alleles), or low-plated fish (one or less *A* or *B* allele) (Hagen and Gilbertson 1973b). Additional models have proposed either epistatic interactions between a single major locus and one modifier locus, or the presence of more than two alternative alleles at the major locus to account for variant results in some populations (Ziuganov 1983; Banbura 1994). All of these models were proposed before the development of genomewide genetic markers for sticklebacks (Peichel et al. 2001) and have never been tested by linkage mapping. In this study, we take advantage of these recently developed tools to examine the genetic basis of variation in lateral plate phenotypes in natural populations of sticklebacks.

## Results

To directly analyze the number and location of genetic loci that control plate phenotypes, we crossed a completely plated marine fish with a low-plated benthic fish from Paxton Lake, British Columbia. Three hundred sixty progeny from a single F2 family (Cross 1) were examined in detail for the pattern, number, and size of lateral plates and then genotyped for the inheritance of different alleles at 160 polymorphic loci distributed across all linkage groups. The segregation of plate phenotypes was compared to the segregation of all genetic markers using quantitative trait loci (QTL) analysis (MapQTL; van Ooijen et al. 2002). Significance thresholds for detecting linkage were chosen using conservative criteria for genomewide linkage mapping in noninbred populations (log likelihood ratio [LOD] score ≥ 4.5; van Ooijen 1999).

**Figure 2:**
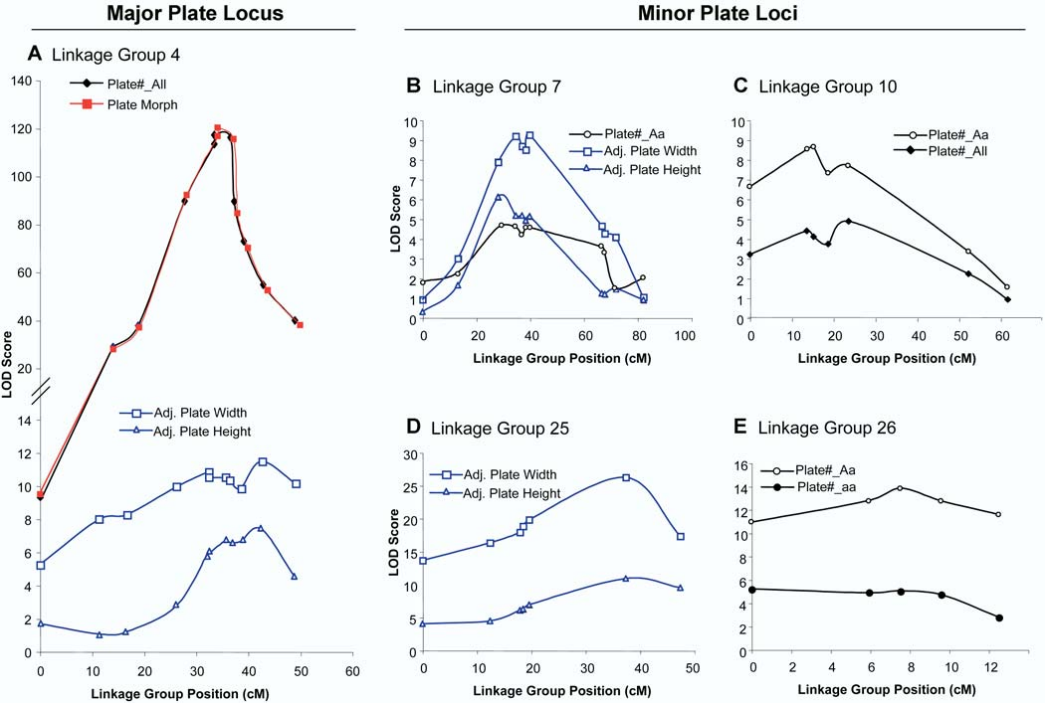
Comparison of QTL Positions for Different Traits LOD scores are shown as a function of genetic distance along different stickleback linkage groups. QTL affecting qualitative plate pattern (red line), total plate number (black lines), or plate size (blue lines) show similar shapes on several linkage groups, suggesting that the same or linked genes control multiple aspects of plate phenotype. Points in LOD plots correspond to the following microsatellite markers from left to right along each linkage group: (A) LG 4: *Pitx2 (Stn220), Stn38, Gac62, Stn42, Gac4174, Stn45, Stn183, Stn46, Stn47, Stn184, Stn39;* (B) LG 7: *Stn70, Stn72, Stn76, Stn71, Stn78, Stn79, Stn75, Stn81, Stn80 Stn82, Pitx1;* (C) LG 10*: Stn119, Stn120, Stn211, Stn121, Stn124, Stn23, Stn125;* (D) LG 25: *Stn212, Stn213, Stn214, Stn215, Stn216, Gac1125, Stn217;* (E) LG 26: *Stn218, Stn219, Bmp6, Stn222, Stn223*. Note that markers *Stn183* and *Stn184* from LG 18 in the Priest Lake cross (Peichel et al. 2001) map together with LG 4 markers in the larger Cross 1.

When plate morph was scored as a qualitative trait, a highly significant QTL on linkage group (LG) 4 was detected (LOD = 117; Table 1 and Figure 2). The genotype of the QTL on LG 4 was highly predictive of the major plate morph that developed in a fish. Almost all fish that carried two alleles from the complete morph grandparent in the LG 4 region (hereafter referred to as *“AA”* animals) showed the complete pattern, whereas fish that carried two alleles from the low morph grandparent in this region (hereafter referred to as *“aa”* animals) showed the low pattern. In contrast, most fish with one allele from the complete grandparent and one allele from the low grandparent (hereafter referred to as *“Aa”* animals) developed as either complete or partial fish (see Figure 1).

**Table 1:**
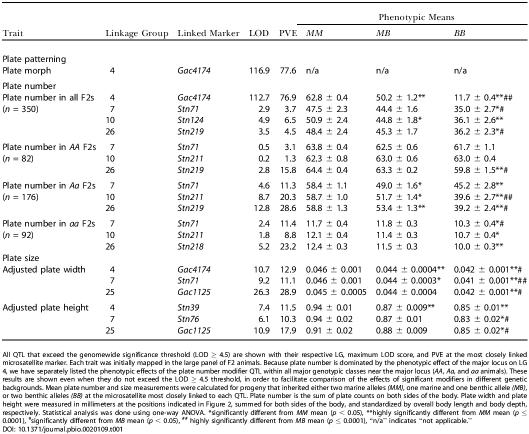
Summary of QTL Affecting Lateral Plate Phenotypes in Cross 1

All QTL that exceed the genomewide significance threshold (LOD ≥ 4.5) are shown with their respective LG, maximum LOD score, and PVE at the most closely linked microsatellite marker. Each trait was initially mapped in the large panel of F2 animals. Because plate number is dominated by the phenotypic effect of the major locus on LG 4, we have separately listed the phenotypic effects of the plate number modifier QTL within all major genotypic classes near the major locus (*AA*, *Aa*, and *aa* animals). These results are shown even when they do not exceed the LOD ≥ 4.5 threshold, in order to facilitate comparison of the effects of significant modifiers in different genetic backgrounds. Mean plate number and size measurements were calculated for progeny that inherited either two marine alleles *(MM)*, one marine and one benthic allele *(MB)*, or two benthic alleles *(BB)* at the microsatellite most closely linked to each QTL. Plate number is the sum of plate counts on both sides of the body. Plate width and plate height were measured in millimeters at the positions indicated in Figure 2, summed for both sides of the body, and standardized by overall body length and body depth, respectively. Statistical analysis was done using one-way ANOVA. *significantly different from *MM* mean (*p* < 0.05), **highly significantly different from *MM* mean (*p* ≤ 0.0001), ^#^significantly different from *MB* mean (*p* < 0.05), ^##^ highly significantly different from *MB* mean (*p* ≤ 0.0001), “n/a” indicates “not applicable.”

**Figure 3:**
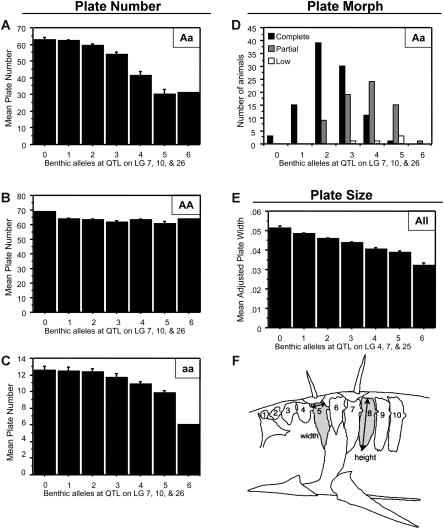
Cumulative Effects of Freshwater Alleles on the Number, Pattern, and Size of Lateral Plates in Cross 1 Increasing the total number of Paxton benthic freshwater alleles at modifier QTL on LGs 7, 10, and 26 significantly reduces plate number in animals with one marine (complete morph) and one Paxton benthic (low morph) allele near the major QTL on LG 4 (*Aa* progeny) (A). The same modifier QTL have little effect on fish with two marine alleles near the major QTL (*AA* animals) (B) and smaller phenotypic effects on animals with two benthic alleles near the major QTL (*aa* animals) (C). Increasing the number of benthic alleles also significantly increases the proportion of *Aa* fish whose overall plate pattern is classified as partial instead of complete (D). (E–F) show plate size effects. Increasing the number of benthic alleles at plate size QTL on LGs 4, 7, and 25 significantly reduces mean plate width of F2 progeny (E). (F) shows the schema of plate size measurements. Lateral plates are shown numbered from anterior to posterior. Error bars in (A–E) represent standard error.

When total plate number was scored, the same major LG 4 chromosome region accounted for more than 75% of the total variance in plate number of F2 fish. Three additional QTL were detected that had significant effects on plate number in *Aa* animals (Table 1; see Figure 2). Increasing the number of benthic alleles at any of the individual modifiers led to a reduction in mean total plate number, even in the heterozygous state (Table 1). Increasing the number of benthic alleles at the three modifiers considered together caused a more than 2-fold reduction in mean plate number of *Aa* animals, largely accounting for whether *Aa* fish developed as either complete, partial, or low morphs (Figure 3A and 3D). Increasing the number of benthic alleles at the same modifier loci also led to a 2-fold reduction in the mean plate number of *aa* animals but had relatively little effect on the plate number of *AA* animals (Figure 3B and 3C). Taken together, these results suggest that at least four different loci influence lateral plate phenotypes in this cross. Homozygosity at the major locus largely determines whether fish develop as low *(aa)* or complete *(AA)* morphs, while the modifier loci affect the actual number of plates, particularly in *Aa* and *aa* animals.

The size of individual lateral plates varies significantly between different stickleback populations (Miller and Hubbs 1969; Avise 1976). Although this trait has not been systematically analyzed in previous stickleback crosses, studies of meristic characters in other vertebrates suggest that the size and number of repeating skeletal elements can be controlled separately (Christians et al. 2003). When height and width of specific plates were analyzed, we detected three QTL that accounted for a significant percentage of plate size variability in the cross (Table 1; see Figure 2). Increasing the number of benthic alleles at these loci led to a progressive reduction in plate size (Figure 3E). Two of the three plate size QTL mapped to the same chromosome regions that also affected plate morph or plate number, suggesting that the pattern, number, and size of plates may be controlled by the same or linked genes on LGs 4 and 7 (see Figure 2). In contrast, the QTL affecting lateral plate size mapped to different locations than most QTL controlling the size of dorsal spine and pelvic structures (Peichel et al. 2001; Shapiro et al. 2004), suggesting that the size of different bones are controlled separately in the stickleback skeleton.

Some of the differences in previously published models of stickleback plate genetics could be due to different genetic mechanisms operating in different populations. To compare the genetic architecture of armor plate patterning in a separate population located over 1300 km from Paxton Lake, we crossed fish from an unusual stickleback population in Friant, California, which is largely dimorphic for complete and low fish with very few partials. A cross between a Friant complete and a Friant low-plated fish resulted in nearly equal numbers of complete and low progeny (see Figure 1, Cross 2), consistent with previous crosses from this population (Avise 1976). Genotyping studies with microsatellite markers linked to the major and minor QTL defined above showed very tight concordance between lateral plate phenotype and genotype near the same major locus on LG 4 that was seen in Cross 1 (LOD = 11.1). All fish with an inferred *Aa* genotype at the major locus on LG 4 were completely plated in this cross, suggesting that *Aa* fish develop more plates in Cross 2 than in Cross 1. This could be due to differences in the dominance relationship of the particular alleles at the LG 4 locus in the Friant population (Avise 1976), or to modification of dominance by the different genetic backgrounds in the two crosses. Although the number of animals in Cross 2 was small, significant differences in the mean total plate count of low fish could also be detected in animals that inherited different alleles at microsatellites linked to two of the modifier QTL detected in Cross 1 (alternative alleles at *Stn210* on LG 7: mean total plate counts 14.9 ± 0.31 vs. 14.0 ± 0.23, *p* = 0.0204; alternative alleles at *Stn219* on LG 26: 14.8 ± 0.26 vs. 13.9 ± 0.31 plates, *p* = 0.0352). Overall, these results suggest that both plate morph and plate number are controlled by similar chromosome regions in different populations.

To further test whether the same major locus on LG 4 controls armor plate reduction in both populations, we carried out genetic complementation crosses between two low female fish from Friant and one low male fish from Paxton Lake. All 84 progeny developed as low morphs, suggesting that the low-plated phenotype in both populations is likely to be due to the same major locus on LG 4.

## Discussion

### QTL Architecture

This study reports the first genomewide linkage mapping of lateral plate phenotypes in crosses between major stickleback plate morphs. Our results confirm previous suggestions that dramatic changes in lateral plate patterning can be controlled by one locus of major effect (Munzing 1959; Avise 1976). This major locus on LG 4 can cause a greater than 5-fold change in total plate number and is sufficient to switch the overall morphology of a fish between the complete, partial, and low-plated states. The dramatic phenotypic effects of this locus likely explain why three types of sticklebacks have long been recognized in natural populations (Cuvier and Valenciennes 1829). Further molecular studies will be required to determine whether there are one or multiple mutations in the LG 4 region that account for the major QTL.

Plate number within the complete, partial, and low morphs also varies between fish from different locations. Previous studies suggest that sticklebacks with small changes in plate number show differential survival when exposed to predators, suggesting that selection may fine tune the exact number of plates in different environments (Hagen and Gilbertson 1973a; Moodie et al. 1973; Reimchen 1992). We have identified three modifier QTL that cause changes in plate number within all morphs but are unlinked to the major locus. The individual phenotypic effects of these QTL can be as small as a single plate per side (Table 1), while the combined mean effects of the QTL can be as large as 15 plates per side (see Figure 3A). The number of modifier QTL is larger than predicted in previous models. We suspect that this is because of the general difficulty of predicting genetic architecture from simple phenotypic ratios of progeny in crosses that are segregating more than one or two genes. The magnitude of the phenotypic effects of the modifiers, their linkage relationships, and interactions with the major locus could not be predicted accurately from previous studies, highlighting the value of genomewide linkage mapping for studying the genetic architecture of major morphological variation in natural populations.

Postglacial freshwater stickleback populations are thought to be derived from completely plated marine ancestors (Bell and Foster 1994). At all of the plate QTL detected in Cross 1, the net effect of the freshwater alleles from the Paxton benthic grandparent is to cause a progressive reduction in the size or number of armor plates (Table 1). All of the QTL that affect plate morph or plate number also have significant effects in the heterozygous state, showing that plate reduction is likely to evolve through semiadditive genetic changes, rather than through purely recessive or purely dominant mutations. Theoretical studies suggest that semiadditive mutations can be fixed more quickly than purely recessive or dominant mutations when they begin at low frequency, although the overall probability of fixation also depends on whether the mutations arise de novo or are originally present in a founder population (Crow and Kimura 1970; Orr and Betancourt 2001). Strong selection on a small number of chromosome regions that have large, semiadditive effects may help explain how dramatic changes in lateral plate patterns have evolved relatively quickly in postglacial stickleback populations.

### Parallel Evolution

Our mapping and complementation results suggest that the same major locus on LG 4 causes major changes in plate pattern in both the Paxton benthic and Friant populations. Phenotypic reduction of lateral plates almost certainly evolved separately in these different locations, given the geographic distance between them (over 1300 km), the presence of completely plated fish in the marine environment separating the sites, and previous studies showing that sticklebacks in nearby lakes have independent mitochondrial haplotypes (Taylor and McPhail 1999). Additional complementation crosses between low-plated fish from Friant and other California populations (Avise 1976; unpublished data), Paxton benthic fish and pelvic-reduced fish from Iceland (Shapiro et al. 2004), and low-plated populations from British Columbia and Japan (Schluter et al. 2004) also produce low-plated progeny. Thus genetic changes at the same major locus may underlie low-plated phenotypes at numerous locations around the world.

The present study provides the first genetic mapping evidence that some of the chromosome regions controlling smaller quantitative variation in plate number may also be used repeatedly in different populations. The QTL on LG 26 in Cross 1 maps to a similar position as a QTL influencing plate number within low morph fish from Priest Lake, British Columbia (Peichel et al. 2001). This QTL is also associated with significant variation in plate number of low morphs of the Friant population (Cross 2), suggesting that this chromosomal region on LG 26 contributes to plate number variation in at least three different populations: Paxton, Priest, and Friant sticklebacks.

Recent studies suggest that the same genes are also used repeatedly when pigmentation and larval cuticle phenotypes have evolved in parallel in different fly populations (Gompel and Carroll 2003; Sucena et al. 2003) or when melanism has evolved independently in birds and mammals (reviewed in Majerus and Mundy 2003). Repeated use of particular genes may thus be a common theme in parallel evolution of major morphological changes in natural populations of both invertebrates and vertebrates.

Why might some genes be used preferentially when similar phenotypes evolve in parallel in wild populations? Alleles that cause plate reduction may already be present at low frequency in marine populations. In that case, parallel phenotypic evolution could occur by repeated selection for the same preexisting alleles in different freshwater locations. Alternatively, some genes may be particularly susceptible to de novo mutations, either because of the size or structure of coding and regulatory regions, or the presence of hotspots for recombination, insertion, or deletion. Finally, only a limited number of either old or new mutations may actually be capable of producing a specific phenotype without also causing deleterious effects on fitness. Mutations with the largest positive selection coefficients will be fixed most rapidly in evolving populations, and this may lead to parallel selection for mutations in the same genes in different populations.

A major goal for future work will be to identify the actual genes and mutations that cause parallel evolution of adaptive traits in wild sticklebacks. This study identifies specific markers that are closely linked to chromosome regions that control the pattern, number, and size of lateral plates. With the recent development of BAC libraries and physical maps of the stickleback genome, it should be possible to use forward genetic approaches to identify the genes responsible for the repeated evolution of major morphological transformations in stickleback armor (Kingsley et al. 2004). Cloning and sequencing of such genes will make it possible to determine the molecular mechanisms that underlie parallel evolution in natural populations and should provide new insight into the nature of genetic, genomic, developmental, and ecological constraints that operate as new characteristics appear during the adaptive evolution of vertebrates.

## Materials and Methods

### 

#### Fish crosses and husbandry

For Cross 1, a wild-caught, completely plated marine female from Onnechikappu stream on the east coast of Hokkaido Island, Japan, was crossed to a wild-caught, low-plated benthic male from Paxton Lake, British Columbia. Both parents showed morphologies typical of the marine and benthic populations at their respective collecting sites. The specific populations were chosen because the large average body size of both parents and the estimated divergence between eastern and western Pacific Ocean fish (Orti et al. 1994) were expected to help maximize the size of the progeny, the number of offspring per clutch, and the informativeness of microsatellites and other markers for genetic mapping. F1 progeny were raised to maturity in 30-gallon aquaria and were mated in pairs. Approximately 2600 F2 progeny were raised to a standard length of greater than 28 mm under the same conditions (30-gallon aquaria in a single 18°C room with 16 hours of light and eight hours of dark per day and twice daily feeding of brine shrimp or frozen blood worms). Although limited phenotypic plasticity has been reported for development of some trophic characters in sticklebacks (Day et al. 1994), previous studies have shown that differences in plate number of wild-caught sticklebacks are stable and reproducible when fish are raised under laboratory conditions (see, for example, Hagen 1967). A total of 360 full siblings from a single F2 family were used for genotypic and phenotypic analysis in this study. For Cross 2, one wild-caught, completely plated female from Friant, California, was crossed to one wild-caught, low-plated male from Friant, California. A total of 58 F1 progeny were raised to a standard length of greater than 28 mm in a ZMOD (Marine Biotech, Beverly, Massachusetts, United States). For the complementation cross, two wild-caught, low-plated females from Friant were crossed to one wild-caught, low-plated benthic male from Paxton Lake, British Columbia. At total of 84 F1 progeny were raised to a standard length of greater than 28 mm in 30-gallon aquaria.

#### Genotyping

Genotyping of microsatellite markers was performed and analyzed essentially as described in Peichel et al. (2001). Some PCR products were analyzed on a 48-capillary array on an ABI3730xl with GeneMapper v3.0 software and GeneScan 500 LIZ (Applied Biosystems, Foster City, California, United States) used as an internal size standard. A total of 160 markers were analyzed in Cross 1, including 144 previously described microsatellite markers (Peichel et al. 2001), the genes *Pitx1*, *Pitx2 (Stn220),* and *Tbx4 (Stn221),* (Shapiro et al. 2004) and 13 new markers: *Bmp6* gene and 12 additional microsatellites *(Stn210–219, 222–223).* A polymorphism within the 3′ UTR of the *Bmp6* gene was genotyped using single strand conformation polymorphism analysis with MDE Gel Solution (BioWhittaker Molecular Applications, Rockland, Maine, United States). PCR bands were visualized using autoradiography. PCR conditions were the same as for the microsatellite markers except 2.5 mM MgCl_2_ and 10% DMSO were used. Primers for *Bmp6* genotyping are: Bmp6F1: 5′ CCCGGTTT*AA*ATCCTCATCC and Bmp6R1: 5′ AGGAGGTGATTGACAGCTCG.

#### Morphological analysis and QTL mapping

Fish were stained with alizarin red to detect skeletal structures as described in Peichel et al. (2001). Lateral plates were counted on both sides of each fish. For QTL mapping, the total plate number of both sides was used. Plate width was measured on the first lateral plate located under the first dorsal spine and above the ascending process of the pelvis. Plate height was measured on the lateral plate posterior to the last plate that is under the second dorsal spine and touching the ascending process. These correspond to plate positions 5 and 8 in previous nomenclature (Reimchen 1983). All measurements were done with Vernier calipers accurate to 0.02 mm and had repeatabilities of 1.1% ± 0.9% (SD)(plate width) and 3.9% ± 2.9% (SD)(plate height). Plate width and height measurements on both sides of the body were summed and standardized by body length and depth, respectively. Similar QTL were detected when residuals from regressions of plate width and height on standard body length and depth were mapped. When raw plate width and height measurements were used, we also detected one additional significant QTL on LG 19 (plate width: LG 19, LOD = 5.42, 7.3 percent variance explained [PVE]; plate height: LG 19, LOD = 7.3, 11.6 PVE). Standard body length itself maps to LG 19 (LOD = 10, 13 PVE). The LG 19 effect on plate size is not significant when plate measurements are normalized by standard body length, suggesting that the LG 19 QTL is a general body size QTL, while the other size QTLs (Table 1; see Figure 2) act on plate size separately from total body size.

All morphological traits in Cross 1 were analyzed with MapQTL 4.0 (van Ooijen et al. 2002) using the same parameters as described by Peichel et al. (2001). Microsatellite markers that were closely linked to QTL detected in Cross 1 were genotyped in all Cross 2 animals (*Gac4174, Stn40,* and *Stn47* on LG 4; *Stn210, Stn71,* and *Stn76* on LG 7; *Stn211* and *Stn121* on LG 10; and *Stn218, Stn219,* and *Stn222* on LG 26). LOD scores between LG 4 markers and the major plate locus in Cross 2 were calculated using Map Manager v2.6.6 (Manly 1993). Mean total plate numbers in low-plated fish that inherited different alleles at microsatellite loci on LGs 4, 7, 10, and 26 were compared using one-way ANOVA (Statview v5.0.1, SAS Institute Inc., Cary, North Carolina, United States).

## Supporting Information

The GenBank accession numbers for the *Bmp6* gene is AY547294 and for the 12 additional new microsatellites *Stn 210–219, 222–223* are BV102488–BV102499.

## Abbreviations

LG: linkage group
LOD: log likelihood ratio
PVE: percent variance explained
QTL: quantitative trait loci
